# *Lactobacillus rhamnosus* Lysate for Metabolic Dysfunction-Associated Steatotic Liver Disease: A Randomized Controlled Trial

**DOI:** 10.3390/medicina62101893

**Published:** 2026-09-30

**Authors:** Maryana Savytska, Yeva Ilkiv, Olena Baka, Elina Christian, Viktoriia Yerokhovych, Dmytro Kyriienko, Tetyana Falalyeyeva, Pavlo Petakh, Iryna Halabitska, Oleksandr Kamyshnyi, Liubov Sichel, Nazarii Kobyliak

**Affiliations:** 1 Physiology Department, Danylo Halytsky Lviv National Medical University, 79010 Lviv, Ukraine; msavytska88@gmail.com; 2 Endocrinology Department, Bogomolets National Medical University, 01601 Kyiv, Ukraine; evailkiv333@gmail.com (Y.I.); elinam@ukr.net (E.C.); korinna.viktoriaer@gmail.com (V.Y.); 3 Center for Innovative Medical Technologies, National Academy of Sciences of Ukraine, 04053 Kyiv, Ukraine; olenabaka@yahoo.com; 4 Kyiv City Clinical Endocrinology Center, 01601 Kyiv, Ukraine; d.v.kirienko@gmail.com; 5 Educational and Scientific Centre “Institute of Biology and Medicine”, Taras Shevchenko National University of Kyiv, 01601 Kyiv, Ukraine; tfalalyeyeva@gmail.com; 6 Medical Laboratory CSD, Scientific, 02000 Kyiv, Ukraine; 7 Department of Biochemistry and Pharmacology, Uzhhorod National University, 88000 Uzhhorod, Ukraine; pavlo.petakh@uzhnu.edu.ua; 8 KSE Health, Aging and Diseases Center, Kyiv School of Economics, 02000 Kyiv, Ukraine; kamyshnyi_om@tdmu.edu.ua; 9 Department of Therapy and Family Medicine, I. Horbachevsky Ternopil National Medical University, 46001 Ternopil, Ukraine; halabitska@tdmu.edu.ua; 10 MirImmunoPharm, 04111 Kyiv, Ukraine; luba@stellarbiotics.com; 11 Stellar Biotics, Rockleigh, NJ 07647, USA

**Keywords:** probiotic derivatives, postbiotics, metabiotics, gut microbiota, MASLD, fatty liver index, hepatic steatosis index, triglyceride-to-glucose index

## Abstract

*Background and Objectives*: Modulation of the gut–liver axis with non-viable microbial components is a promising approach to correct metabolic disorders. The aim of the study was to evaluate the short-term efficacy and safety of the postbiotic lysate *Lactobacillus rhamnosus* DV-NRRL B-68023 on non-invasive measures of hepatic steatosis, transaminase activity, and anthropometric parameters in patients with MASLD. *Materials and Methods*: In a randomized, double-blind, placebo-controlled trial (NCT06352697), 52 adults were assigned 1:1 to receive the postbiotic Del-Immune V^®^ Extra (100 mg twice daily; *n* = 26) or placebo (*n* = 26) for 3 months followed by a 3-month follow-up without treatment. The registered co-primary endpoints were FLI, HSI, and TyG. Additionally, transaminases, lipid parameters, hs-CRP, and body composition indices were assessed. *Results*: In the ITT analysis (*n* = 52), FLI showed a nominal time-by-treatment interaction (*p* = 0.049), with a between-group difference in change at month 3 of −7.20 points (95% CI: −12.95 to −1.46). HSI and TyG did not differ significantly between groups. In exploratory ITT analyses, waist circumference showed a significant time-by-treatment interaction (*p* = 0.003), whereas the interaction for visceral fat was not significant (*p* = 0.079). ALT and AST decreased significantly within the postbiotic group, but the corresponding time-by-treatment interactions were not significant (*p* = 0.340 and *p* = 0.111, respectively). Lipid parameters and hs-CRP did not differ significantly between groups. All adverse events were mild and self-limiting. *Conclusions*: Three months of *Lactobacillus rhamnosus* lysate DV-NRRL B-68023 was well tolerated. Among the three registered co-primary surrogate indices, only FLI showed a nominal between-group difference; HSI and TyG did not. An exploratory reduction in waist circumference was observed, whereas the ALT and AST changes were not significantly different from placebo. These findings warrant confirmation in larger studies using direct liver assessments and longer follow-up.

## 1. Introduction

Metabolic-associated steatohepatitis (MASLD) encompasses a wide spectrum of liver lesions, from simple steatosis to metabolic-associated steatohepatitis (MASH), fibrosis, cirrhosis, and MASLD-associated hepatocellular carcinoma (MASLD-HCC) [1,2]. MASLD is currently one of the most common liver diseases worldwide. The global burden of this disease has increased significantly between 1990 and 2023, posing significant economic and health-related social challenges [3]. Given the increasing prevalence of modifiable risk factors, the incidence and long-term burden of MASLD worldwide are expected to continue to increase through 2050 [3].

The pathogenesis of MASLD is heterogeneous and has traditionally been explained by the concept of “multiple hits,” in which genetic predisposition interacts with environmental factors to lead to hepatic lipid accumulation, insulin resistance, and obesity [4,5]. Genome-wide association studies have identified key single-nucleotide polymorphisms, including in the PNPLA3, TM6SF2, MBOAT7, and GCKR genes, that may significantly increase the risk of developing MASLD [6]. Although the disease is closely associated with components of the metabolic syndrome, such as obesity and type 2 diabetes [7,8], MASLD is also increasingly being diagnosed in individuals of normal or low body weight. This condition has been described as “underweight liver disease” [9].

At the cellular level, MASLD begins with the accumulation of fat in the liver, when it exceeds 5% of liver mass [10]. This contributes to the formation of reactive oxygen species (ROS), impaired mitochondrial function, and lipotoxicity [11,12]. The influx of free fatty acids increases oxidative stress and activates liver immune cells. As a result, the production of pro-inflammatory cytokines, including IL-6, IL-1β, and TNF-α, is increased, and triglyceride metabolism is dysregulated [13,14].

The gut microbiota plays an important role in maintaining metabolic balance through the so-called “gut–liver axis” [15,16]. A diet high in fat and refined carbohydrates can cause gut dysbiosis, reducing the number of beneficial bacteria and the overall diversity of the gut microbiota and viruses [17]. Disruption of the microbiota can weaken the intestinal epithelial barrier, which facilitates the penetration of bacterial endotoxins and other microbial components through the portal vein to the liver [18]. This maintains chronic inflammation in the liver and causes metabolic changes, forming a vicious cycle that contributes to the progression of MASLD [19].

Modification of the intestinal microbiota with pre-, pro-, para- and postbiotics is considered a promising therapeutic approach [20,21,22,23]. Traditional probiotics can improve intestinal barrier function and inhibit the growth of pathogenic microorganisms, but their use is associated with certain limitations in terms of viability, safety and stability, especially in patients at high risk of complications [24]. Therefore, in recent years, interest in postbiotics and metabiotics, which contain non-viable microbial components or their metabolic products, has increased [25]. Postbiotics containing cell wall fragments and nucleic acids have several advantages, including a well-defined chemical structure, longer shelf life, better pharmacokinetics, and a favorable safety profile [26,27].

Lactic acid bacteria (LAB), including Lactobacillus rhamnosus, and their lysates have demonstrated anti-inflammatory, antioxidant, and lipid-modulating properties. Their use may contribute to reductions in triglyceride accumulation, dyslipidemia, and HOMA-IR levels [28,29,30]. The DV-NRRL B-68023 lysate was selected because prior experimental work with *L. rhamnosus* lysates and prior clinical use of the same Del-Immune V^®^ product suggested immunometabolic activity and acceptable tolerability [23,27,28,29,30]. However, clinical data on the efficacy of such products specifically in patients with MASLD remain limited.

Therefore, the aim of this study was to conduct a randomized, placebo-controlled clinical trial to evaluate the short-term efficacy and safety of the postbiotic lysate “Del-Immune V^®^ Extra” (*Lactobacillus rhamnosus* DV-NRRL B-68023) in patients with MASLD. The effect of the study product was assessed using non-invasive biochemical indices of steatosis, transaminase activity, lipid parameters, markers of chronic systemic inflammation, and anthropometric measures.

## 2. Materials and Methods

### 2.1. Ethics Statement

This double-blind, placebo-controlled, parallel-group randomized controlled trial ran at the Kyiv City Clinical Endocrinology Centre, Ukraine, with recruitment starting in May 2024. The local Ethics Committee approved the protocol (No. 2/2023), and the trial followed the Declaration of Helsinki. The trial was registered at ClinicalTrials.gov (NCT06352697; DELI_MASLD Study). Before enrollment, all participants received information about the study objectives and procedures and provided written informed consent to participate.

### 2.2. Inclusion Criteria

The inclusion criteria were adult participants (ages 18–70), the presence of MASLD according to new EASL–EASD–EASO criteria [31], and a diagnosis of steatotic liver disease (SLD) on the basis of abdominal ultrasonography results. With respect to 4 known criteria (hepatorenal echo contrast, liver brightness, deep attenuation, and vascular blurring), the participants were required to have hepatorenal contrast and liver brightness to be given a diagnosis of SLD; a fatty liver index (FLI) greater than 60; a BMI of 25–39.9 kg/m^2^; and an aspartate transaminase (AST) and alanine transaminase (ALT) level ≤ 3× the upper limit of normal. Written informed consent was also obtained.

### 2.3. Exclusion Criteria

The exclusion criteria were as follows: recent hepatitis or a positive screening test for hepatitis B (hepatitis B virus surface antigen) or hepatitis C (hepatitis C antibody); alcohol abuse (>20 g/day (2 standard drinks) in women or >30 g/d (3 drinks) in men over a two-year period); drug-induced liver disease, Wilson’s disease, hereditary deficiency of antitrypsin-1 and idiopathic hemochromatosis; a history of decompensated liver disease, including ascites or variceal bleeding; regular use of agents with gut microbiota modulation activity (antibiotic, pro, pre, post- or synbiotic supplements, etc.) within 3 months prior to enrollment; allergy to probiotics or their components; use of agents such as vitamin E, omega-3 fatty acids or medications with evidence for effects on MASLD (pioglitazone, GLP-1 analogs, dipeptidyl peptidase IV inhibitors, ursodeoxycholic acid); a history of bariatric surgery or significant weight loss (>5% body weight) or rapid weight loss (>1.6 kg/week) within 6 months prior to enrollment; uncontrolled cardiovascular or respiratory disease; decompensated liver disease, including ascites, encephalopathy or variceal bleeding, active malignancy, or chronic infections; and participants who had a severe course of COVID-19.

### 2.4. Study Design

This RCT involved 52 adults diagnosed with MASLD who satisfied all study criteria. The study comprised an initial 7-day screening window, a 12-week active intervention period, and a subsequent 3-month post-treatment follow-up. To reduce the influence of abrupt lifestyle changes on metabolic parameters, participants underwent a 14-day pre-treatment stabilization run-in after providing informed consent. During this period, participants received individualized nutritional counseling focused on a lifestyle-modification diet consistent with National Cholesterol Education Program (NCEP) recommendations. Pre-existing doses of glucose-lowering medications were kept unchanged, and participants were instructed to perform 60 min of low-intensity physical activity daily. Following stabilization, participants were allocated 1:1 to receive Del-Immune V^®^ Extra or an identical placebo for 12 weeks. An independent statistician generated the allocation sequence electronically via www.randomization.com, with balance by age, sex, and baseline disease severity. The principal investigator recruited participants, and allocation was implemented using sequentially numbered, opaque, sealed envelopes opened only after baseline data entry and confirmation of eligibility. Active and placebo capsules were iden-tical in appearance, smell, and packaging. Participants, staff dispensing the capsules, la-boratory personnel, and the statistician were blinded until database closure. Lifestyle recommendations were reinforced through monthly telephone contacts, participant diaries, and scheduled visits. Diet and physical activity adherence were assessed by self-report; caloric intake and physical activity were not objectively quantified, and group-specific quantitative lifestyle-adherence variables were not retained in the analysis dataset.

An adverse event (AE) was considered any unwanted symptom or condition that occurred during the study, regardless of whether it was related to the study product. Adverse events were classified by severity as mild, moderate, and severe. Mild was considered to be short-term and well-tolerated symptoms that did not affect normal daily activities. Moderate adverse events caused noticeable discomfort and partially limited daily activities. Severe adverse events significantly impaired the participant’s condition and completely prevented the participant from performing normal daily activities. If a minor AE occurred, patients retained the autonomy to either continue or cease product intake, though they were still encouraged to attend all subsequent follow-up evaluations. Individuals who experienced severe gastrointestinal disorders, such as severe diarrhea or vomiting, systemic infection, or required systemic antibiotic interventions during the study timeframe were instructed to discontinue the study product but were encouraged to complete follow-up assessments; available outcome data were retained in the ITT analysis.

At the end of week 12, adherence to the assigned study product was assessed by comparing returned capsule counts with participant diary entries. Adherence was prespecified as intake of at least 85% of assigned doses. Participants below this threshold or requiring prohibited antibiotic therapy were classified as having protocol deviations; however, participants with available outcome data were retained in the ITT analyses in their randomized groups.

### 2.5. Supplements

During the study, participants received Del-Immune V^®^ Extra, containing a lyophilized enzymatic cell lysate with DNA fragments of *Lactobacillus rhamnosus* DV-NRRL B-68023, at a dose of 100 mg twice daily, or matching placebo capsules. The 100 mg twice-daily regimen was selected on the basis of prior clinical use of the same study product [27]. Del-Immune V^®^ Extra was produced by MirImmunoPharm LLC (Kyiv, Ukraine) in cooperation with Stellar Biotics, LLC (Rockleigh, NJ, USA). Published characterization of the same *L. rhamnosus* DV lysate reports approximately 100 mg/g protein, 118 mg/g DNA, 15–18 mg/g muramyl peptides, pH 6.5–7.5 for a 1% suspension, and moisture ≤5% [32]. The placebo contained microcrystalline cellulose and was identical in color, weight, appearance, and packaging. The active study product and matching placebo were supplied in kind by MirImmunoPharm LLC. Detailed batch-specific quality-control documentation was maintained by the manufacturer and was not part of the clinical study dataset available for the present analysis. Therefore, additional technical details regarding batch-release testing and confirmation of bacterial inactivation could not be reported in the current manuscript.

### 2.6. Outcomes Assessment and Measurement

After providing informed consent, patients submitted fasting serum samples that were immediately frozen at −20 °C. The relevant clinical and demographic information was collected for each individual. Laboratory tests were performed in a certified medical laboratory, CSD, Kyiv, Ukraine.

After providing informed consent, participants provided fasting serum samples that were processed according to the laboratory protocol and stored at −20 °C until analysis. Relevant clinical and demographic information was collected for each participant. Laboratory testing was performed at the certified Medical Laboratory CSD, Kyiv, Ukraine, using the laboratory’s validated standard operating procedures and routine internal quality-control processes. Laboratory measurements were performed in a certified medical laboratory according to its standardized routine procedures. Detailed platform-, reagent-, and lot-specific analytical information was outside the scope of the clinical dataset used for the present analysis.

The three outcomes registered as primary at ClinicalTrials.gov were the fatty liver index (FLI), hepatic steatosis index (HSI), and triglyceride-to-glucose (TyG) index. The FLI was calculated via a formula that incorporates BMI, waist circumference (WC), triglyceride level, and GGT level [33]:

FLI = (e^(0.953 × ln(triglycerides) + 0.139 × BMI + 0.718 × ln(GGT) + 0.053 × waist circumference − 15.745))/(1 + e^(0.953 × ln(triglycerides) + 0.139 × BMI + 0.718 × ln(GGT) + 0.053 × waist circumference − 15.745)) × 100

To calculate the HSI, a formula that includes clinical and laboratory parameters such as body mass index (BMI) and aspartate aminotransferase (AST) and alanine aminotransferase (ALT) levels was used, with adjustments for diabetes and sex [34]:

HSI = 8 × ALT/AST + BMI (+2 if type 2 diabetes is yes, +2 if female)

The TyG index is calculated on the basis of fasting blood triglyceride and glucose levels via the following formula [35]:

TyG = ln [Fasting triglyceride (mg/dL) × Fasting glucose (mg/dL)]/2

FLI, HSI, and TyG were evaluated as co-primary surrogate outcomes, in accordance with the trial registration, and were analyzed without hierarchical prioritization. Given the assessment of three co-primary endpoints, the corresponding *p*-values should be interpreted with due consideration of multiplicity. As FLI is a composite index incorporating BMI, waist circumference, triglycerides, and GGT, changes in FLI are inherently correlated with changes in its constituent anthropometric and biochemical variables and therefore should not be interpreted as independent evidence of a treatment effect on hepatic steatosis.

Registered secondary outcomes included waist circumference, BMI, visceral fat, AST, ALT, GGT, total cholesterol, triglycerides, LDL-C, HDL-C, VLDL-C, hs-CRP, and IL-6. Complete longitudinal measurements of LDL-C, HDL-C, VLDL-C, and IL-6 were not available across all prespecified study visits; therefore, these variables were not included in the final analysis. The secondary outcome analyses reported in the present study comprised the variables with complete longitudinal assessment.

All patients underwent anthropometry, in which body height (BH) was measured accurately to 0.001 m and body weight (BW) was measured accurately to 0.001 kg via medical scales. BMI was calculated via the Quetelet formula: BMI = BW/BH^2^.

The waist circumference (WC) was measured via flexible tape at the level of the belly button, with an accuracy of 0.001 m.

Visceral fat content was determined by bioimpedance measurement using the Tanita Scale BC-601 electronic body composition analyzer (Tanita Corporation, Tokyo, Japan).

### 2.7. Sample Size Calculation

The study was designed to enroll 52 participants (26 per group), providing a minimum of 48 evaluable participants (24 per group) after allowing for potential attrition. The sample size target was informed by the magnitude of change in FLI observed in the randomized trial by Kobyliak et al. (2018) [36] together with the anticipated feasibility of recruitment within the study setting. The study was primarily designed to evaluate longitudinal changes in the prespecified co-primary surrogate outcomes. Analyses of secondary outcomes and subgroup effects were considered exploratory.

### 2.8. Statistical Analysis

Statistical processing of the data was carried out using SPSS software, version 20.0 (SPSS Inc., Chicago, IL, USA), and GraphPad Prism, version 11.0 (GraphPad Software, La Jolla, CA, USA), with a two-sided *p*-value of less than 0.05 considered statistically significant. The baseline quantitative variables are expressed as mean values with standard deviations (M ± SD), while categorical data are shown as percentages. The distribution of continuous variables was examined using the Kolmogorov–Smirnov test together with Q–Q plots and histograms. Changes between baseline and post-intervention measurements within each group were analyzed using paired *t*-tests. Categorical variables were compared using the chi-square test or Fisher’s exact test, depending on the expected cell frequencies.

Because the trial had three timepoints (baseline, month 3, and month 6), two-way repeated-measures ANOVA (RM-ANOVA) was used to evaluate longitudinal trajectories. All 52 randomized participants had observed outcome data at the three study visits and were analyzed in their randomized groups (ITT). The model included time, treatment group, and the time-by-treatment interaction; no additional covariates were included. Mauchly’s test was used to assess sphericity, with the Greenhouse–Geisser correction applied when necessary. Bonferroni-adjusted post hoc pairwise comparisons were used within each RM-ANOVA model. Changes were calculated as follow-up minus baseline and are presented with 95% confidence intervals (CIs). To complement interaction *p*-values, unadjusted between-group differences in mean change (postbiotic minus placebo) and 95% CIs were calculated from participant-level change scores using Welch–Satterthwaite standard errors and are reported at month 3 and month 6. Because the three registered primary outcomes were analyzed in parallel and no separate prespecified multiplicity procedure could be documented, primary *p*-values are interpreted as nominal.

Secondary outcomes were analyzed in the ITT population using the same observed-data framework and were considered exploratory. No imputation was required because outcome data were available for all randomized participants at the scheduled visits. Secondary *p*-values were treated as nominal because no formal multiplicity correction was prespecified across these outcomes. Exploratory treatment-by-T2D and treatment-by-sex interaction analyses for change in FLI were also performed and were considered hypothesis-generating.

## 3. Results

### 3.1. Efficacy

The recruitment phase of this randomized controlled trial (RCT) lasted from May to September 2024 (Figure 1). At the initial screening stage, 128 individuals were examined, of whom 76 were excluded from further participation. In particular, 52 candidates did not meet the established inclusion and exclusion criteria, and 24 individuals did not provide informed consent to participate in the study.

As a result, 52 participants who met the criteria were included in the study, after which they were randomized in a 1:1 ratio to two parallel groups: the postbiotic therapy group (Del-Immune V^®^ Extra, *n* = 26) and the control group (placebo, *n* = 26).

At the initial stage, demographic characteristics and main clinical indicators were similar in both groups. No statistically significant differences between the groups were found (*p* > 0.05; Table 1).

All 52 randomized participants (26 per group) were included in the intention-to-treat (ITT) analyses according to their original treatment allocation. Complete follow-up data were available for all participants, and no imputation was required. Four participants experienced protocol deviations related to study-product exposure: two in the Del-Immune V^®^ Extra group and two in the placebo group. As outcome data remained available at the scheduled assessment timepoints, these participants were retained in the ITT efficacy analyses.

Study-product adherence ≥85% was achieved by 24/26 participants (92.3%) in the Del-Immune V^®^ Extra group and 25/26 participants (96.2%) in the placebo group. Four protocol deviations were recorded: antibiotic therapy (*n* = 1) and discontinuation of the study product for personal reasons (*n* = 1) in the Del-Immune V^®^ Extra group, and antibiotic therapy (*n* = 2) in the placebo group.

During the 6-month study period, all participants received standard medical care, MASLD management recommendations, and the same lifestyle-modification instructions. Diet and physical activity were reviewed through participant diaries and monthly con-tacts, but adherence was not objectively quantified and group-specific numerical lifestyle-adherence data were not available for analysis. Accordingly, the lifestyle program is considered a standardized co-intervention and a potential contributor to change in both groups. Baseline clinical variables did not differ significantly between groups (*p* > 0.05). Among the three co-primary outcomes, FLI showed a nominal time-by-treatment interaction (*p* = 0.049; Table 2). At month 3, FLI changed by −6.28 points (95% CI: −11.31 to −1.25) in the postbiotic group and by 0.92 points (95% CI: −2.07 to 3.92) in the placebo group; the between-group difference in change was −7.20 points (95% CI: −12.95 to −1.46). At month 6, the between-group difference in change was −3.00 points (95% CI: −6.68 to 0.68).

HSI showed a significant overall time effect (*p* = 0.001), with within-group decreases at month 3 in both the placebo and Del-Immune V^®^ Extra groups. However, the time-by-treatment interaction was not significant (*p* = 0.737), indicating no evidence that the HSI trajectory differed between groups. The between-group difference in change was −0.52 points (95% CI: −1.84 to 0.81) at month 3 and −0.43 points (95% CI: −2.12 to 1.26) at month 6. TyG did not show a significant time-by-treatment interaction (*p* = 0.261; Table 2, Figure 2C). The between-group difference in change was −0.11 (95% CI: −0.24 to 0.03) at month 3 and −0.05 (95% CI: −0.18 to 0.08) at month 6.

Changes in transaminase activity are presented in Figure 3. In the exploratory ITT analysis of secondary outcomes, waist circumference showed a significant time-by-treatment interaction (*p* = 0.003; Table 3, Figure 4C). At month 3, the between-group difference in change was −1.40 cm (95% CI: −2.26 to −0.55), and at month 6 it was −1.62 cm (95% CI: −2.50 to −0.73). Although statistically detectable, the absolute magnitude was modest and its clinical relevance requires confirmation. The overall time-by-treatment interaction for visceral fat was not statistically significant (*p* = 0.079; Figure 4D). The between-group difference in change was −0.56 percentage points (95% CI: −1.46 to 0.35) at month 3 and −0.98 percentage points (95% CI: −1.85 to −0.12) at month 6.

Body weight, BMI, total cholesterol, triglycerides, and hs-CRP did not show statistically significant time-by-treatment interactions (*p* > 0.05). ALT and AST decreased within the Del-Immune V^®^ Extra group at month 3 (ALT: −5.88 U/L, 95% CI: −10.27 to −1.49; *p* = 0.011; AST: −2.78 U/L, 95% CI: −4.74 to −0.81; *p* = 0.007), but the between-group interaction tests were not significant (ALT *p* = 0.340; AST *p* = 0.111). The between-group differences in change at month 3 were −3.82 U/L (95% CI: −8.96 to 1.32) for ALT and −2.11 U/L (95% CI: −4.83 to 0.62) for AST; at month 6 they were −2.31 U/L (95% CI: −9.40 to 4.78) and 0.96 U/L (95% CI: −3.00 to 4.91), respectively. Thus, the within-group reductions in transaminases were not statistically different from placebo.

**Figure 3:**
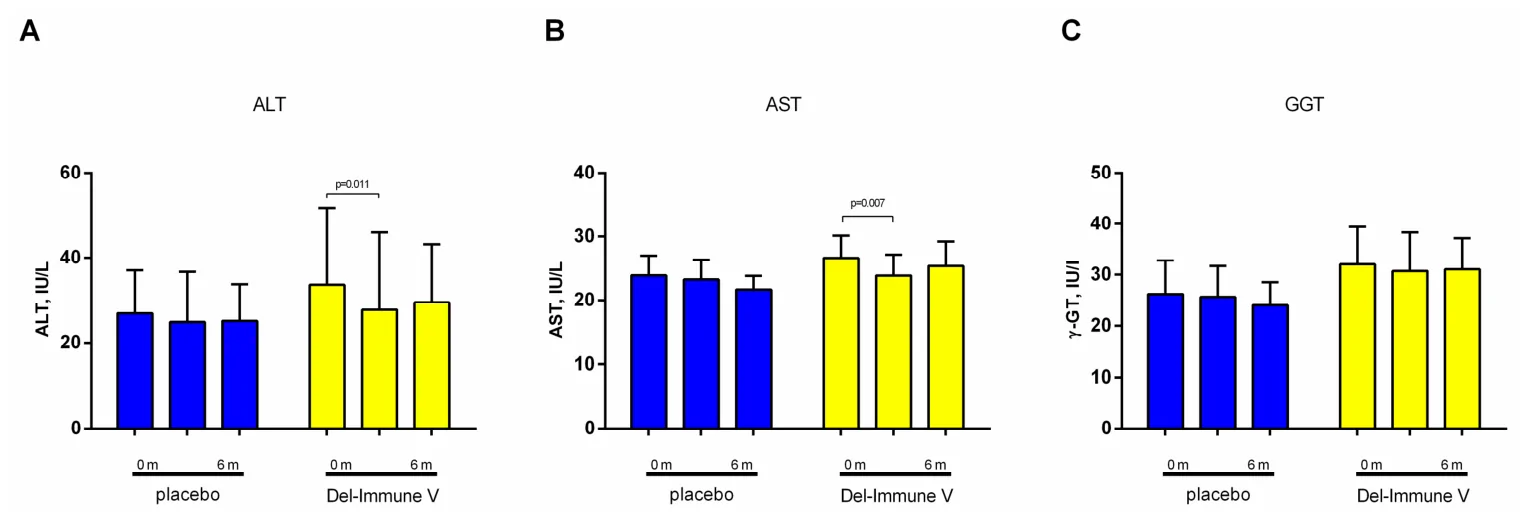
Secondary outcome analysis with a focus on transaminase activity changes ((**A**)—ALT; (**B**)—AST; (**C**)—γ-GT). Data presented as M ± SD.

**Figure 4:**
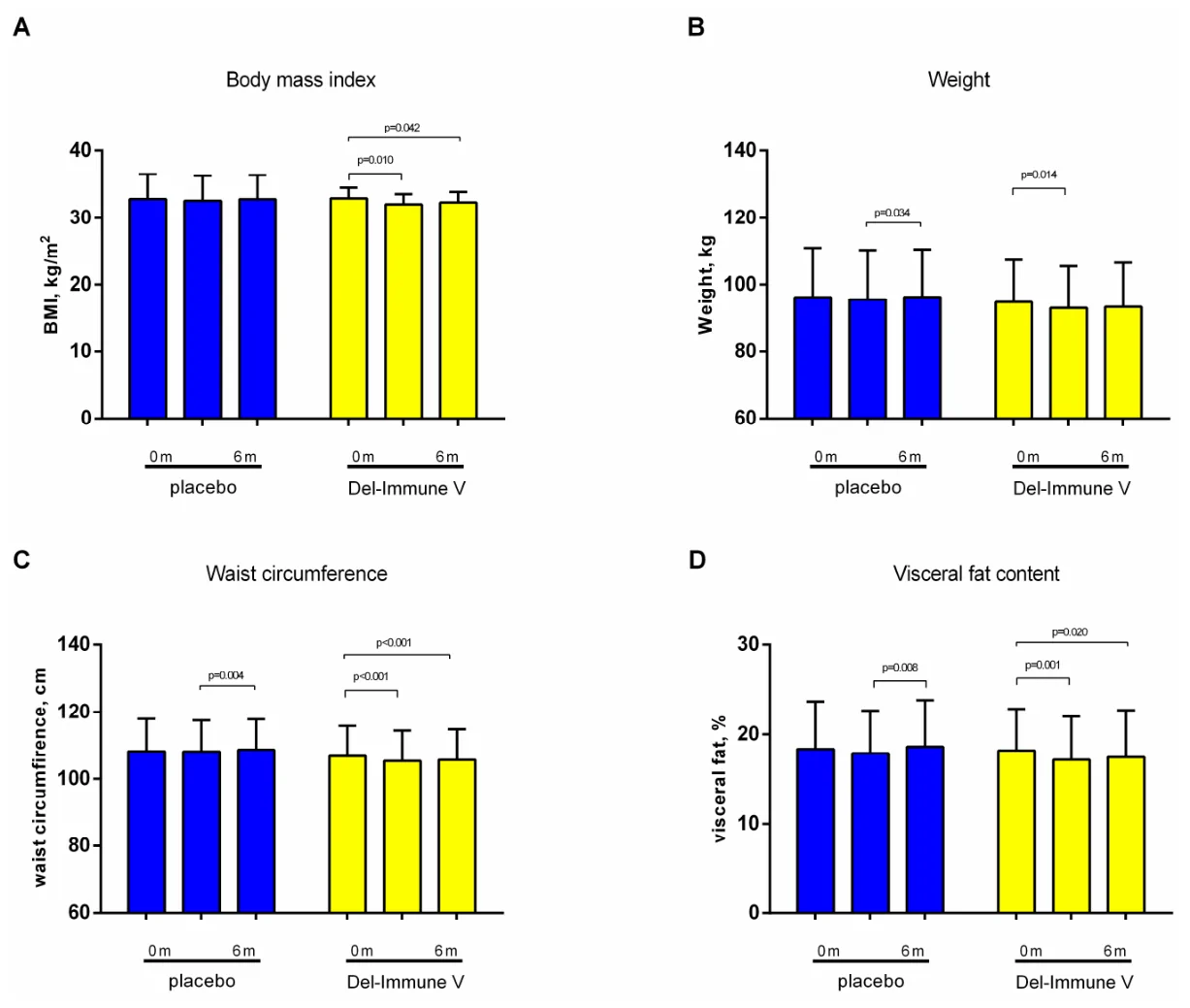
Secondary outcome analysis with a focus on changes in anthropometric parameters ((**A**)—BMI; (**B**)—body weight; (**C**)—WC; (**D**)—visceral fat content). Data presented as M ± SD.

Total cholesterol, triglycerides, and the systemic inflammatory marker hs-CRP (reported in mg/L) showed no significant time-by-treatment interactions throughout the 6-month study period (*p* > 0.05; Table 3, Figure 5). The month-3 between-group difference in hs-CRP change was −0.42 mg/L (95% CI: −2.14 to 1.29). Exploratory interaction analyses did not identify statistically significant effect modification of the month-3 FLI treatment effect by T2D status (interaction *p* = 0.415) or sex (interaction *p* = 0.441).

### 3.2. Safety

Regarding the safety and tolerability profiles, both the active postbiotic and the placebo formulations were exceptionally well accepted, with no severe adverse events (SAEs) documented throughout the entire RCT. All registered AEs were mild in intensity, temporary, and resolved spontaneously without requiring clinical intervention or leading to treatment discontinuation. In the Del-Immune V^®^ Extra group, four mild adverse events (15.4%) were recorded. Two participants experienced abdominal pain and bloating, one had constipation, and one had transient diarrhea. In the placebo group, three adverse events (11.5%) were recorded: one participant had nausea, one had heartburn, and one had a transient headache. There was no statistically significant difference in the frequency of adverse events between the groups (*p* = 0.685). Thus, the postbiotic demonstrated good tolerability. During the next 3-month follow-up after the end of treatment, there were no new complaints or adverse events in participants in either group.

## 4. Discussion

Although MASLD remains strongly associated with obesity, recent epidemiological evidence suggests that hepatic steatosis is increasingly being detected in individuals of normal weight [36,37,38,39,40]. The mainstay of treatment for MASLD is lifestyle modification, including dietary modification, reduction in excessive caloric intake, and adequate physical activity [41]. At the same time, there is growing interest in additional pharmacological and metabolic approaches aimed at reducing hepatic steatosis and its associated metabolic disorders [42,43]. In this context, increasing attention is being paid to biotics as a possible additional therapeutic approach. The use of traditional probiotics may be beneficial in steatotic liver disease, as they contribute to the restoration of the normal composition of the intestinal microbiota, reduce systemic inflammation, and regulate lipid and glucose metabolism [44]. Postbiotics—comprising functional non-viable microbial cells, cell constituents, or metabolites—offer distinct clinical advantages in metabolic disorders [45]. Because they eliminate the requirement for microbial viability, postbiotics possess superior stability under diverse physiological conditions, exhibit a commendable safety profile even in immunocompromised states, and carry a negligible risk of systemic infection or bacteremia [46].

MASLD also has clinically relevant extrahepatic implications. In particular, MASLD/NAFLD and chronic kidney disease (CKD) frequently coexist and share metabolic and inflammatory pathways, including insulin resistance, visceral adiposity, oxidative stress, lipid dysregulation, endothelial dysfunction, and chronic low-grade inflammation [32,47,48,49]. This overlap supports viewing MASLD as a systemic cardiometabolic disorder rather than an isolated liver condition, although the present trial did not assess renal outcomes.

In this double-blind RCT, we evaluated a 3-month intervention with the *L. rhamnosus* DV-NRRL B-68023 lysate (Del-Immune V^®^ Extra). Among the three registered co-primary surrogate outcomes, FLI showed a nominally significant time-by-treatment interaction (*p* = 0.049), whereas HSI (*p* = 0.737) and TyG (*p* = 0.261) did not differ between groups. The month-3 between-group difference in FLI change was −7.20 points (95% CI: −12.95 to −1.46), but the difference was no longer statistically significant at month 6. Because all participants received the same lifestyle intervention and lifestyle adherence was not objectively quantified, changes in both groups may partly reflect this co-intervention. The borderline FLI result and multiplicity across co-primary outcomes warrant cautious interpretation [50].

The exploratory ITT analysis showed a statistically different trajectory for waist circumference (*p* = 0.003), with a month-3 between-group difference of −1.40 cm (95% CI: −2.26 to −0.55). The overall interaction for visceral fat was not significant (*p* = 0.079), and neither body weight nor BMI differed significantly between groups. The absolute waist-circumference difference was modest, and the study was not designed to establish a minimum clinically important difference. Moreover, because FLI directly incorporates waist circumference, BMI, triglycerides, and GGT, the FLI and waist-circumference findings are mathematically related and should not be interpreted as independent evidence of separate hepatic and adiposity effects.

Preclinical work by Rodrigues e-Lacerda et al. showed that gut bacteria-derived postbiotic site-specific immunomodulators can attenuate steatosis, inflammation, and fibrosis in experimental MASLD [22]. Such findings provide a mechanistic rationale for studying postbiotics but do not establish that the clinical changes observed here were mediated by direct hepatoprotective or tissue-specific pathways. The mean age of participants in the present trial was approximately 54 years, broadly comparable with middle-aged adult metabolic-disease cohorts in prior *L. rhamnosus* studies [27,29,30]. The 100 mg twice-daily dose and 3-month treatment duration also match prior clinical use of Del-Immune V^®^ Extra [27], whereas studies using other strains or preparations have differed in dose, du-ration, and target population. These differences limit direct cross-study comparisons.

ALT and AST decreased significantly from baseline within the postbiotic group during treatment, but the corresponding between-group effects were not statistically significant. Accordingly, these within-group changes cannot be interpreted as evidence of a treatment-specific hepatoprotective effect. Transaminase values moved toward baseline after treatment cessation, but in the absence of a significant between-group effect these findings should not be used to infer that prolonged administration or repeated treatment courses are required.

Immunomodulation remains a biologically plausible mechanism through which postbiotics may influence the gut–liver axis [51]. Structural bacterial components, including peptidoglycans and teichoic acids, may interact with innate immune receptors and modulate epithelial-barrier integrity and inflammatory signaling [52,53,54], while preclinical studies of Lactobacillus-derived postbiotics have demonstrated effects on lipogenic and inflammatory pathways [55,56]. In the present study, however, hs-CRP did not differ significantly between groups, suggesting that the observed clinical effects were not accompanied by a detectable change in this systemic inflammatory marker. Given that hs-CRP primarily reflects systemic inflammation, it may not fully capture localized immune activity within the gut–liver axis.

The study product was well tolerated, but the efficacy findings should be interpreted within several limitations: the modest sample size, short follow-up, use of surrogate indices rather than direct quantitative liver imaging or histology, lack of objective quantitative assessment of diet and physical activity adherence, incomplete collection of several originally registered secondary biomarkers (LDL-C, HDL-C, VLDL-C, and IL-6), and incomplete trial-specific documentation of product batch quality-control procedures. The exploratory anthropometric findings and nominal FLI result require confirmation in larger multicenter trials with prespecified multiplicity control, direct liver assessments, and longer follow-up.

## 5. Conclusions

In this randomized, double-blind, placebo-controlled study, 3 months of *Lactobacillus rhamnosus* lysate DV-NRRL B-68023 (Del-Immune V^®^ Extra) was well tolerated. Among the three registered co-primary surrogate indices, FLI showed a nominally significant time-by-treatment interaction, whereas HSI and TyG did not differ significantly between groups. In exploratory ITT analyses, waist circumference decreased more in the postbiotic group, although the absolute difference was modest and FLI partly incorporates waist circumference. ALT and AST decreased within the postbiotic group, but these changes were not significantly different from placebo. The findings support further investigation of this postbiotic in adequately powered studies using direct liver assessments rather than a recommendation for clinical use.

## Figures and Tables

**Figure 1:**
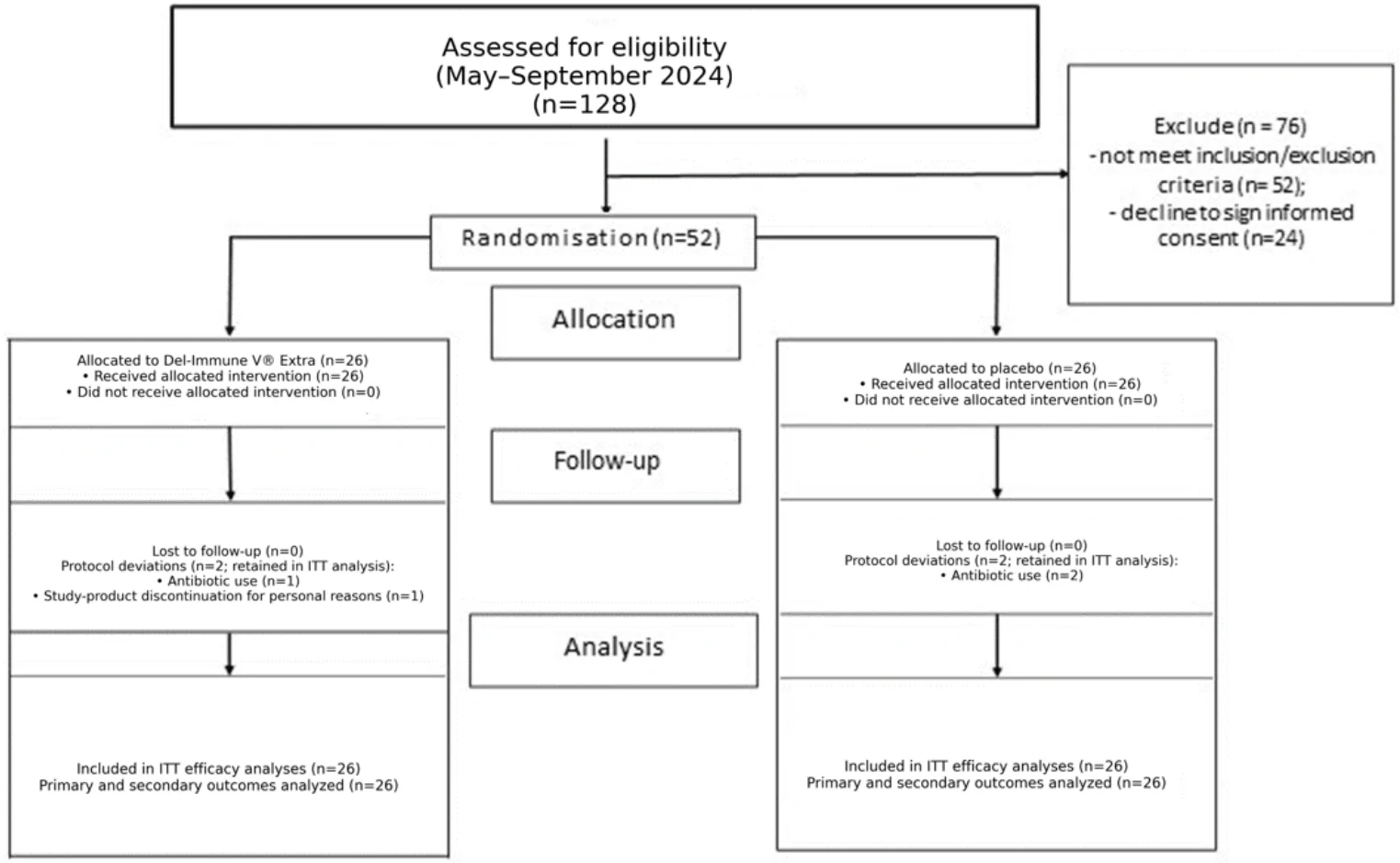
Consolidated Standards of Reporting Trials (CONSORT) flow chart trial protocol.

**Figure 2:**
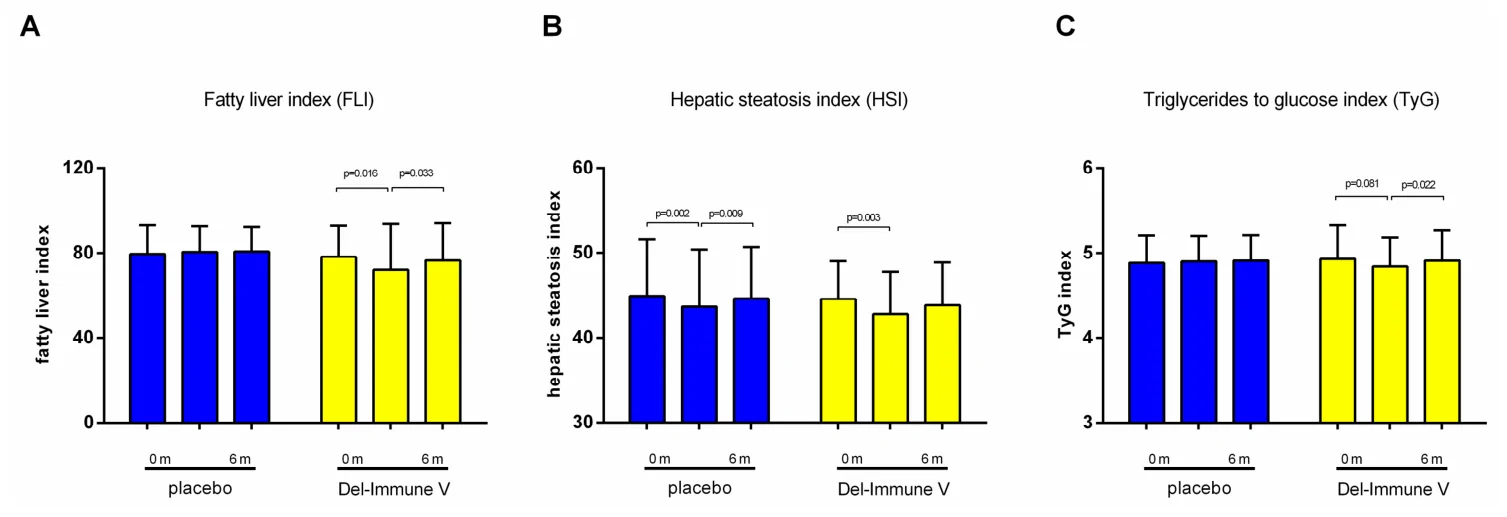
Primary outcomes analysis with accents on changes in hepatic steatosis indices ((**A**)—FLI; (**B**)—HSI; (**C**)–TyG). Data presented as M ± SD.

**Figure 5:**
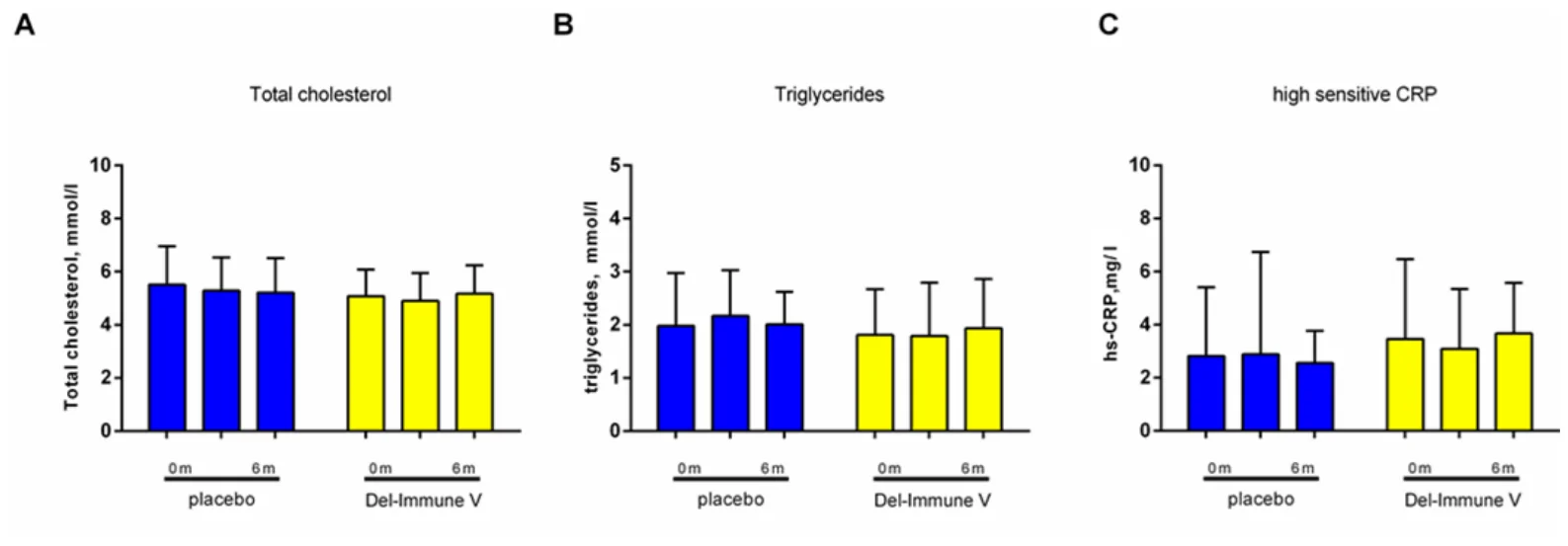
Secondary outcome analysis with a focus on changes in lipids and chronic systemic inflammation markers ((**A**)—total cholesterol; (**B**)—triglycerides; (**C**)—hs-CRP). Data presented as M ± SD.

**Table 1:** Baseline clinical parameters in examined patients (M ± SD or %).

Parameter	Placebo Group (*n* = 26)	Del-Immune (*n* = 26)	*p*
**Age, years**	53.42 ± 14.11	54.15 ± 13.98	0.852
**T2D, % (*n*)**	46.2 (12)	53.8 (14)	0.579
**T2D duration, years**	15.58 ± 8.08	15.14 ± 10.20	0.905
**Women, % (*n*)**	57.7 (15)	50.0 (13)	0.578
**Metformin, % (*n*)**	34.6 (9)	34.6 (9)	0.999
**Insulin, % (*n*)**	15.4 (4)	19.2 (5)	0.714

**Table 2:** Intergroup analysis of primary outcomes.

Parameters	Baseline	p1	Mean Absolute Changes from Baseline		p2	
			∆ 3 Months	∆ 6 Months	Time Effect	Time × Treatment Effect
**FLI**					0.103	0.049
Placebo	79.5 ± 13.4		0.92 (−2.07 to 3.92)	1.19 (−1.50 to 3.88)		
Postbiotic	78.23 ± 14.47	0.744	−6.28 (−11.31 to −1.25)	−1.8 (−4.45 to 0.83)		
Between-group difference (postbiotic–placebo), 95% CI			−7.20 (−12.95 to −1.46)	−3.00 (−6.68 to 0.68)		
**HSI**					0.001	0.737
Placebo	44.92 ± 6.67		−1.27 (−2.05 to −0.50)	−0.30 (−1.27 to 0.67)		
Postbiotic	44.55 ± 4.44	0.813	−1.79 (−2.90 to −0.68)	−0.73 (−2.16 to 0.69)		
Between-group difference (postbiotic–placebo), 95% CI			−0.52 (−1.84 to 0.81)	−0.43 (−2.12 to 1.26)		
**TyG index**					0.342	0.261
Placebo	4.88 ± 0.32		0.02 (−0.07 to 0.11)	0.03 (−0.07 to 0.13)		
Postbiotic	4.92 ± 0.39	0.629	−0.09 (−0.19 to 0.01)	−0.02 (−0.11 to 0.07)		
Between-group difference (postbiotic–placebo), 95% CI			−0.11 (−0.24 to 0.03)	−0.05 (−0.18 to 0.08)		

p1, difference between baseline group means. Baseline values are presented as M ± SD. Changes are calculated as follow-up minus baseline and presented with 95% CI. p2, p-values from repeated-measures ANOVA, comprising the main effect of time and the time × treatment interaction. The time effect tests overall change across the three visits, whereas the time × treatment interaction tests whether longitudinal trajectories differ between randomized groups. No additional covariates were included in the RM-ANOVA model; Greenhouse–Geisser correction was applied if sphericity was violated, and Bonferroni adjustment was used for post hoc pairwise comparisons within each model.

**Table 3:** Intergroup analysis of secondary outcomes.

Parameters	Baseline	p1	Mean Absolute Changes from Baseline		p2	
			∆ 3 Months	∆ 6 Months	Time Effect	Time × Treatment Effect
**ALT, IU/L**					0.012	0.340
Placebo	26.93 ± 10.09		−2.06 (−4.94 to 0.81)	−1.80 (−4.76 to 1.15)		
Postbiotic	33.64 ± 18.15	0.106	−5.88 (−10.27 to −1.49)	−4.11 (−10.67 to 2.43)		
Between-group difference (postbiotic–placebo), 95% CI			−3.82 (−8.96 to 1.32)	−2.31 (−9.40 to 4.78)		
**AST, IU/L**					0.045	0.111
Placebo	23.92 ± 7.66		−0.67 (−2.66 to 1.31)	−2.22 (−4.68 to 0.23)		
Postbiotic	26.66 ± 8.60	0.230	−2.78 (−4.74 to −0.81)	−1.27 (−4.48 to 1.94)		
Between-group difference (postbiotic–placebo), 95% CI			−2.11 (−4.83 to 0.62)	0.96 (−3.00 to 4.91)		
**γ-GT, IU/L**					0.639	0.753
Placebo	26.24 ± 16.22		−0.58 (−3.07 to 1.89)	−2.20 (−5.80 to 1.40)		
Postbiotic	32.08 ± 18.11	0.226	−1.36 (−6.24 to 3.50)	−1.00 (−6.98 to 4.97)		
Between-group difference (postbiotic–placebo), 95% CI			−0.78 (−6.16 to 4.60)	1.20 (−5.65 to 8.04)		
**BMI, kg/m^2^**					0.003	0.318
Placebo	32.85 ± 3.64		−0.28 (−0.75 to 0.18)	−0.05 (−0.67 to 0.57)		
Postbiotic	32.95 ± 3.87	0.927	−0.88 (−1.53 to −0.23)	−0.63 (−1.24 to −0.02)		
Between-group difference (postbiotic–placebo), 95% CI			−0.60 (−1.39 to 0.18)	−0.58 (−1.43 to 0.27)		
**Weight, kg**					0.010	0.364
Placebo	96.26 ± 14.65		−0.69 (−1.95 to 0.55)	0.01 (−1.51 to 1.53)		
Postbiotic	94.86 ± 12.40	0.712	−1.83 (−3.27 to −0.39)	−1.51 (−3.05 to 0.02)		
Between-group difference (postbiotic–placebo), 95% CI			−1.14 (−3.00 to 0.72)	−1.53 (−3.64 to 0.59)		
**WC, cm**					0.001	0.003
Placebo	108.13 ± 9.86		−0.01 (−0.59 to 0.55)	0.48 (−0.25 to 1.21)		
Postbiotic	106.67 ± 9.16	0.582	−1.42 (−2.09 to −0.75)	−1.13 (−1.66 to −0.60)		
Between-group difference (postbiotic–placebo), 95% CI			−1.40 (−2.26 to −0.55)	−1.62 (−2.50 to −0.73)		
**Visceral fat, %**					0.002	0.079
Placebo	18.34 ± 5.34		−0.50 (−1.21 to 0.21)	0.23 (−0.39 to 0.85)		
Postbiotic	18.17 ± 4.68	0.902	−1.05 (−1.64 to −0.46)	−0.75 (−1.37 to −0.12)		
Between-group difference (postbiotic–placebo), 95% CI			−0.56 (−1.46 to 0.35)	−0.98 (−1.84 to −0.12)		
**Total cholesterol, mmol/L**					0.419	0.349
Placebo	5.51 ± 0.45		−0.22 (−0.67 to 0.23)	−0.29 (−0.81 to 0.21)		
Postbiotic	5.08 ± 1.00	0.221	−0.17 (−0.58 to 0.23)	0.08 (−0.27 to 0.44)		
Between-group difference (postbiotic–placebo), 95% CI			0.04 (−0.55 to 0.64)	0.38 (−0.23 to 1.00)		
**Triglycerides, mmol/L**					0.724	0.243
Placebo	1.98 ± 0.99		0.18 (−0.16 to 0.54)	0.02 (−0.36 to 0.41)		
Postbiotic	1.81 ± 0.85	0.511	−0.02 (−0.29 to 0.25)	0.12 (−0.10 to 0.35)		
Between-group difference (postbiotic–placebo), 95% CI			−0.21 (−0.65 to 0.23)	0.10 (−0.35 to 0.54)		
**hs-CRP, mg/L**					0.939	0.542
Placebo	2.82 ± 2.58		0.06 (−1.55 to 1.67)	−0.25 (−1.14 to 0.62)		
Postbiotic	3.46 ± 3.00	0.417	−0.36 (−0.99 to 0.27)	0.21 (−0.63 to 1.05)		
Between-group difference (postbiotic–placebo), 95% CI			−0.42 (−2.14 to 1.29)	0.47 (−0.72 to 1.66)		

p1, difference between baseline group means. Baseline values are presented as M ± SD. Changes are calculated as follow-up minus baseline and presented with 95% CI. p2, p-values from repeated-measures ANOVA, comprising the main effect of time and the time × treatment interaction. p(time) tests the overall effect of time across the three visits, and p(time × treatment) tests whether longitudinal trajectories differ between randomized groups. No additional covariates were included in the RM-ANOVA model; Greenhouse–Geisser correction was applied if sphericity was violated, and Bonferroni adjustment was used for post hoc pairwise comparisons within each model. Secondary analyses are exploratory and *p*-values are nominal.

## Data Availability

The data presented in this study are available from the corresponding author upon reasonable request. The data are not publicly available because they contain information that could compromise the privacy of the research participants.
